# Accidental Puncture of a Dialysis Catheter into the Brachiocephalic Trunk

**DOI:** 10.1055/s-0040-1715466

**Published:** 2020-12-23

**Authors:** Mohamad Syafeeq Faeez Md Noh

**Affiliations:** 1 Department of Imaging, Faculty of Medicine and Health Sciences, Universiti Putra Malaysia, Serdang, Selangor, Malaysia; 2 Department of Radiology, Universiti Putra Malaysia (UPM) Teaching Hospital, Serdang, Selangor, Malaysia

**Keywords:** central venous cannulation, computed tomography angiography, imaging, vascular injury, sternotomy

## Abstract

A 22-year-old male with underlying end-stage renal failure was referred to our center for a malpositioned dialysis catheter. Imaging showed the tip of the catheter to be placed in the brachiocephalic trunk; the patient was, however, asymptomatic. Surgical removal of the malpositioned catheter followed, with no postoperative complications.

A 22-year-old male with underlying end-stage renal failure was referred to our center for a malpositioned dialysis catheter. He has been on regular continuous ambulatory peritoneal dialysis via a Tenckhoff's catheter; however, suspicion of a blocked catheter necessitated its removal.

Right internal jugular dialysis catheter (size, 16 French) was inserted via an anatomical landmark technique.

After insertion, pulsatile flow, as well as an appearance of oxygenated blood, raised suspicion of an inadvertent arterial injury. No obvious hematoma was clinically observed, and the patient did not report neurological or obstructive symptoms. He was clinically stable.

An urgent computed tomography angiography (CTA) revealed the catheter puncturing through the medial aspect of the right internal jugular vein, and entering the right subclavian artery (black arrow, Fig. 1 ), and passing retrograde into the brachiocephalic trunk, where the tip was seen (white arrow, Fig. 1 ). Minimal hematoma was observed. A cardiothoracic surgery referral was sought.

**Fig. 1:**
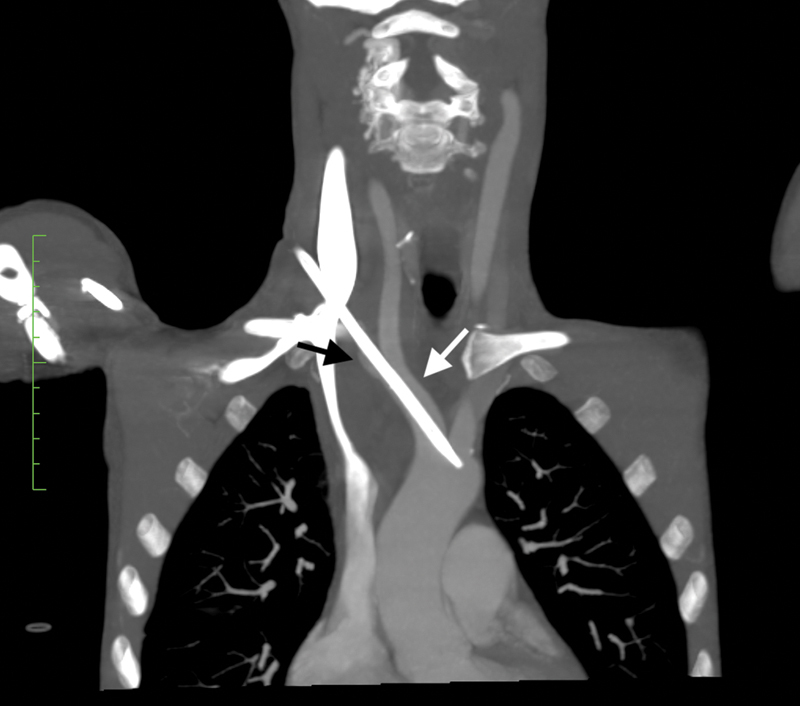
Computed tomography angiography image, in coronal section, showing the course of the malpositioned dialysis catheter, after puncturing through the internal jugular vein (medial aspect). The catheter is seen to course through the right subclavian artery (black arrow), with the tip within the brachiocephalic trunk (white arrow).

A partial sternotomy, exploration of the subclavian and jugular vessels and surgical catheter removal were done. Intraoperatively, the catheter was seen exiting the right internal jugular vein at its medial aspect, penetrating the right subclavian artery at its superior aspect. The catheter could be felt within the right subclavian artery and the brachiocephalic trunk. The catheter was then removed, and the right subclavian artery was clamped both proximally and distally. The injured vessels were repaired using prolene 5/0. No overt hematoma was seen.

The patient recovered well postoperatively and was discharged on day 3. Postoperative radiograph did not show any complications ( Fig. 2 ).

**Fig. 2:**
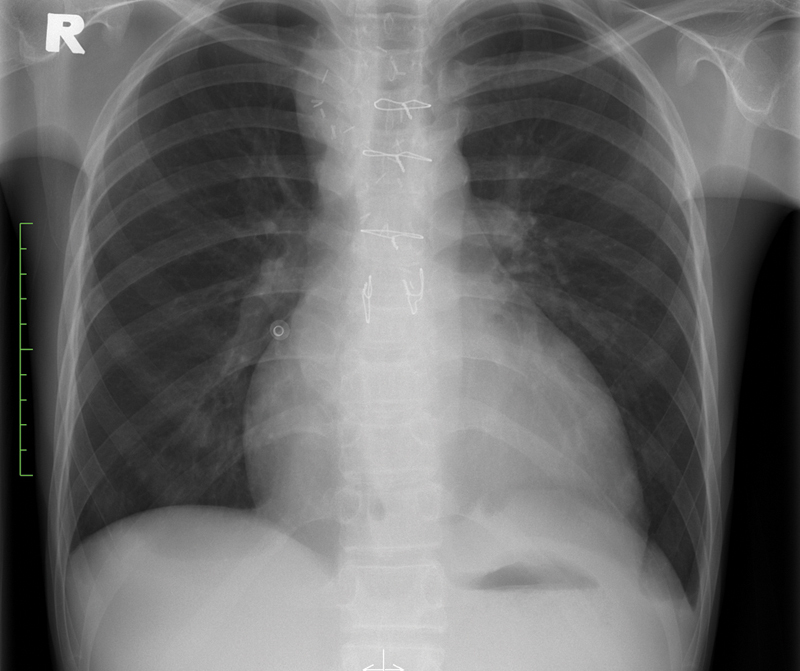
Postoperative frontal chest radiograph on outpatient follow-up was normal.

Central venous catheters are used routinely in clinical practice for a multitude of reasons as follows: venous access, prolonged antibiotics administration, nutritional support, as well as in managing perioperative fluid status. However, insertion of these catheters poses associated risks, among them being inadvertent arterial puncture, which can occur in up to 6% of patients when using external landmark techniques. 1 Complications that may accompany arterial injuries include hematoma formation, stroke, fistula, development of pseudoaneurysms, and death.

Central venous catheter insertion via ultrasound guidance has been shown in multiple studies to reduce catheter-related complications by up to 71%. 2 However, this technique, albeit preferable, is not routinely practiced at this patient's referring center.

When arterial injuries do occur, management has to be tailored on a case-by-case basis, taking into account the expertise and services available in a particular institution. The catheter is secured to prevent movement or dislodgment, followed by cross-sectional imaging via CT or magnetic resonance imaging, to better delineate the position of the catheter, guiding subsequent treatment approach.

In our center, the cardiothoracic surgery service is readily available, hence the surgical approach was conducted.

An alternative technique would be via an endovascular approach, utilizing covered stents, balloon tamponade, or vascular closure devices. 3
